# Mucin 4 mutation is associated with tumor mutation burden and promotes antitumor immunity in colon cancer patients

**DOI:** 10.18632/aging.202756

**Published:** 2021-03-14

**Authors:** Linglong Peng, Yang Li, Haitao Gu, Ling Xiang, Yongfu Xiong, Rong Wang, He Zhou, Jijian Wang

**Affiliations:** 1 Department of Gastrointestinal Surgery, The Second Affiliated Hospital of Chongqing Medical University, Chongqing 400010, China; 2 Department of Clinical Nutrition, The Second Affiliated Hospital of Chongqing Medical University, Chongqing 400010, China; 3 Department of Hepatobiliary Surgery, The Affiliated Hospital of North Sichuan Medical College, Sichuan 637000, China; 4 Department of Oncology, The Cancer Center of the Fifth Affiliated Hospital of Sun Yat-Sen University, Zhuhai 519000, China; 5 Department of Gastrointestinal Surgery, The Affiliated Hospital of North Sichuan Medical College, Sichuan 637000, China

**Keywords:** colon cancer, mucin 4, tumor mutation burden, tumor-infiltrating immune cells, prognosis

## Abstract

At present, immunotherapy is widely used for different mismatch repair (dMMR) or highly microsatellite instability (MSI-H) colorectal cancer patients, and tumor mutation burden (TMB) is a valuable independent predictor of response to immunotherapy. However, specific gene mutations and their relationship with TMB and tumor-infiltrating immune cells in colon cancer remains unclear. In the present study, we analyzed somatic mutation data of colon cancer from The Cancer Genome Atlas (TCGA) and International Cancer Genome Consortium (ICGC) datasets, and found that 17 frequently mutated genes were occurred in both cohorts, including APC, TP53, TNN, KRAS, MUC16, MUC4 (mucin 4), SYNE1, FLG, FAT4, OBSCN, FAT3, RYR2, PIK3CA, FBXW7, DNAH11, MUC5B and ZFHX4. Interestingly, only MUC4 mutation was associated with higher TMB and patient clinical prognosis among the 17 mutated genes. Moreover, according to gene set enrichment analysis (GSEA) and the CIBERSORT algorithm, we revealed that MUC4 mutation activated signaling pathways involved in the immune system and enhanced the antitumor immune response. In conclusion, MUC4 may have important clinical implications for immune therapy of colon cancer.

## INTRODUCTION

Colon cancer is a common cause of morbidity and mortality worldwide [1]. There were an estimated 101420 new cases of colon cancer in the US in 2019, and an estimated 44180 patients died from colorectal cancer in the same year [2]. For resectable nonmetastatic colon cancer, the preferred treatment method is colectomy plus regional lymph node dissection [3]. Unfortunately, approximately 50%-60% of patients diagnosed with colon cancer develop metastases, and of these, 80% to 90% are nonresectable liver metastases with poor prognosis [4, 5]. In recent years, colon cancer patients have benefited significantly from 5-Fu- and oxaliplatin-based chemotherapy regimens, and newly developed targeted drugs also prolong the survival time of patients with advanced colon cancer [6, 7]. However, recurrence and metastasis remain major problems in colon cancer and are often the ultimate causes of death.

Currently, colon cancer has shifted from the inherent treatment mode of "surgery mainly, chemoradiotherapy supplemented" to the treatment concept of precision and individual, and immunotherapy is receiving increasing attention [8]. In 2015, Le et al. found that metastatic colorectal cancer (mCRC) with either different mismatch repair (dMMR) or highly microsatellite instability (MSI-H) molecular phenotypes can significantly benefit from the immune checkpoint inhibitor (ICPI) programmed death ligand 1 (PD-L1) monoclonal antibody pembrolizumab [9]. Although subsequent studies have further expanded the immunotherapy indications for MSI-H/dMMR colorectal cancer from the original posttreatment of metastatic disease to first-line treatment and neoadjuvant therapy for early disease [10, 11], the effective population of immunotherapy is still limited to this specific group. Currently, immunohistochemical detection of PD-L1 has been widely used to screen patients with colorectal cancer who can benefit from immunotherapy [12]. However, the tumor tissue microenvironment can interfere with PD-L1 expression, and the relationship between the expression of PD-L1 in colorectal cancer and the efficacy of immunotherapy is not exact [13]. Moreover, the response rate of MSI-H colorectal cancer patients to ICPI is also variable, and tumor responders have more somatic mutations and higher neoantigen load than nonresponders [14], indicating the need for additional predictive biomarkers.

Tumor mutant burden (TMB) is a biomarker reflecting somatic mutation and is expected to pave the way for tumor immunotherapy to enter the era of precision medicine [15]. During the occurrence and development of cancer, a mass of somatic mutations can produce neoantigens, which increased tumor immunogenicity and thereby activated immune recognition system [16]. The high production of neoantigens is associated with enhanced checkpoint blocking responses, and along with the recognition of neoantigens, the activity of T cells against the tumor was increased by immune system thereby enhancing the efficacy of ICPI [17–19]. TMB is an emerging independent biomarker that can be used to stratify the possible response of patients to ICPC [20]. A previous study found that among the patients with high TMB lung cancer, the response rate for ICPI was higher than that in the patients with low TMB expression, and the clinical outcome was significantly improved, suggesting that high TMB was positively correlated with the efficacy of immunotherapy [21]. In addition, Zaravinos et al. have reported that colon cancer cells possess a higher mutation load and neoepitope load, which drives the immune system to fight against tumors [22]. However, the changes of specific gene mutations and their relationship with TMB and tumor-infiltrating immune cells in colon cancer remains unclear. Therefore, the aim of our study is to identify mutated genes using TCGA and ICGC colon cancer samples, and to further explore the association of mutated genes with TMB and patient outcome and infiltrating immune cells.

## RESULTS

### Somatic mutation characteristics in colon cancer

**Figure 1:**
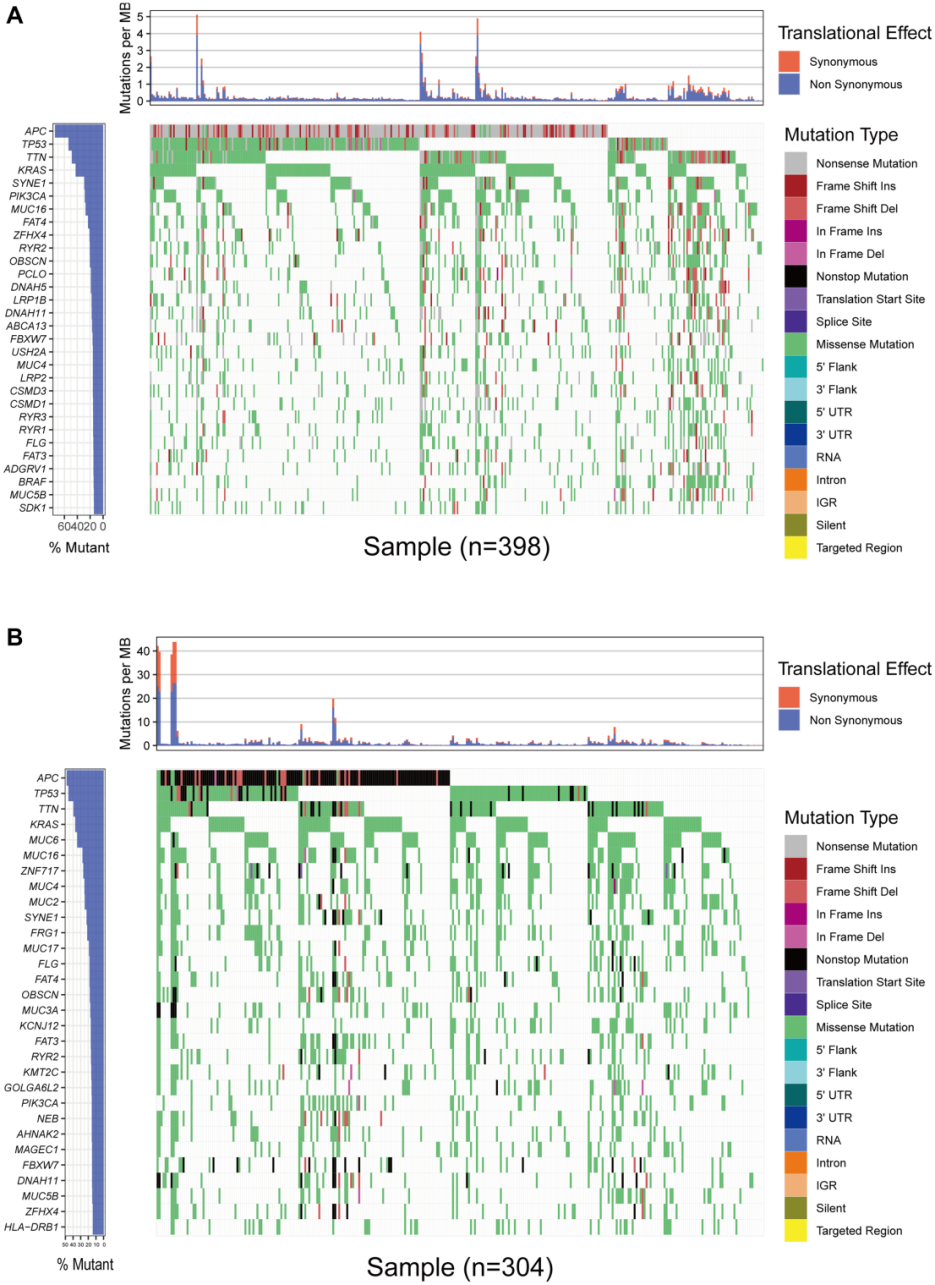
**Overview of frequently mutated genes in colon cancer.** (**A**) Waterfall plot shows the frequently mutated genes in colon cancer from TCGA database. The left panel shows mutation frequency, and genes are ordered by their mutation frequencies. The right panel presents different mutation types. (**B**) Waterfall plot displaying the frequently mutated genes in colon cancer from the ICGC cohort. The left panel shows the genes ordered by their mutation frequencies. The right panel presents different mutation types.

We first downloaded the mutation data of 398 American colon cancer samples from TCGA, and the cumulative mutations frequency in each gene was counted and sorted in decreasing order. The top 30 frequently mutated genes with high mutation frequency and the pattern of somatic mutation for the top 30 genes are illustrated in Figure 1A. The top 5 mutated genes were *APC* (74%), *TP53* (54%), *TTN* (48%), *KRAS* (43%), and *SYNE1* (29%). Similarly, the top 30 mutated genes were also identified in Chinese patients from ICGC database. As shown in Figure 1B, missense mutation was occurred commonly in Chinese patients, and *APC* (49%), *TP53* (46%), *TTN* (39%), *KRAS* (37%), and *MUC6* (35%) had the top 5 mutation frequency among Chinese patients.

### Gene mutations associated with TMB

**Figure 2:**
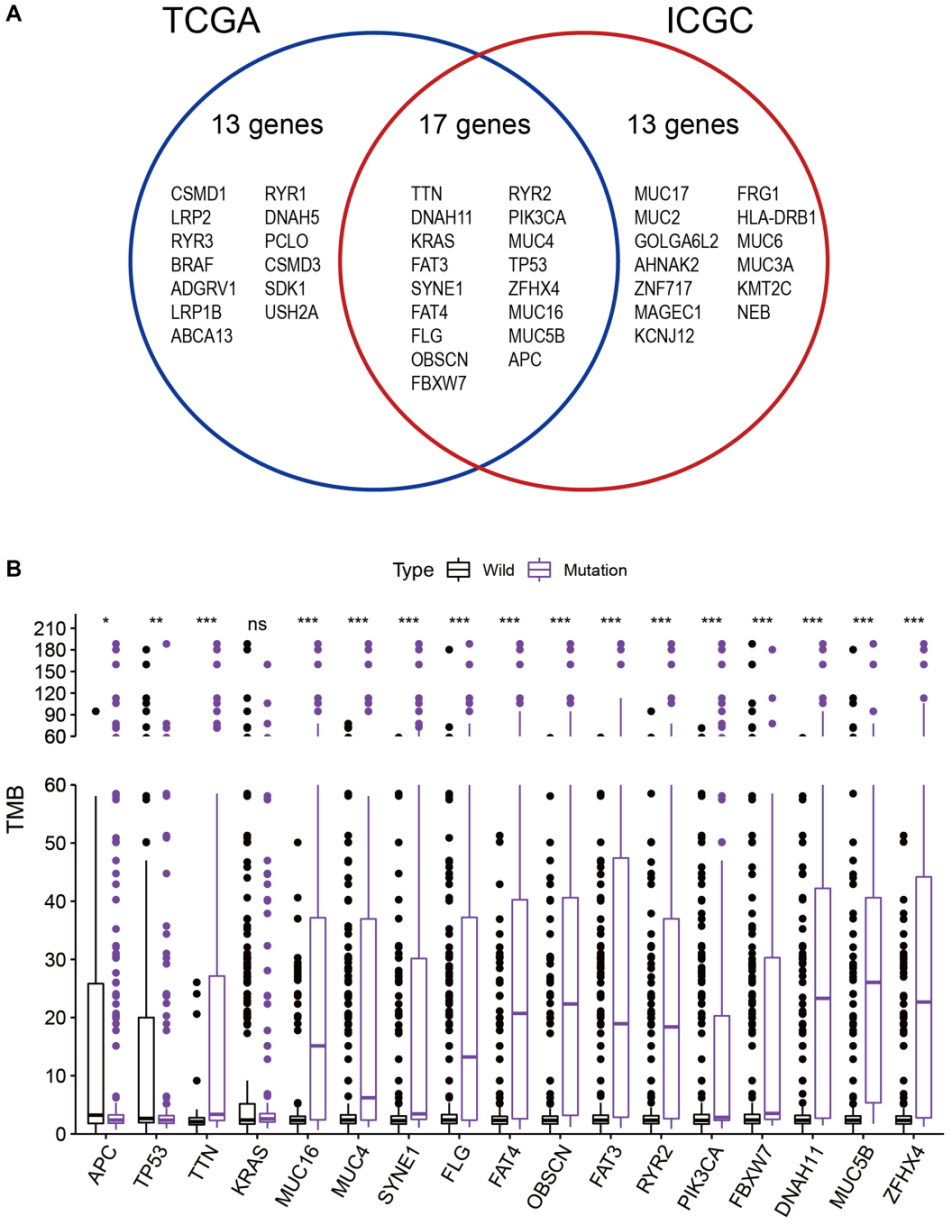
**Gene mutations are associated with TMB.** (**A**) Venn diagram shows 17 frequently mutated genes covered by both the TCGA and ICGC cohorts. (**B**) Sixteen genes with high mutation frequency are associated with a higher TMB. ^*^p < 0.05; ^**^p < 0.01; ^***^p < 0.001; ns: no significance.

Next, to obtain the genes that are commonly mutated in both TCGA and ICGC databases. we intersected the genes with the top 30 mutation rates in the two cohorts. As shown in Figure 2A, the intersection genes with high mutations were *APC*, *TP53*, *TNN*, *KRAS*, *MUC16*, *MUC4*, *SYNE1*, *FLG*, *FAT4*, *OBSCN*, *FAT3*, *RYR2*, *PIK3CA*, *FBXW7*, *DNAH11*, *MUC5B* and *ZFHX4*. To further investigated whether these 17 commonly mutated genes were associated with TMB, colon cancer patients from TCGA cohort were classified into wild group and mutation group based on the 17 gene mutation status. In addition, TMB expression for each TCGA sample was calculated, and the median value of TMB is 9.95 per Mb (0.05-188.32 per Mb). With combining analysis of the data of gene mutation matrix and TMB expression matrix, we found that TMB value in mutation group of all the other 16 genes except *KRAS* was significantly changed compared with wild group (Figure 2B).

### *MUC4* mutation associated with prognosis

**Figure 3:**
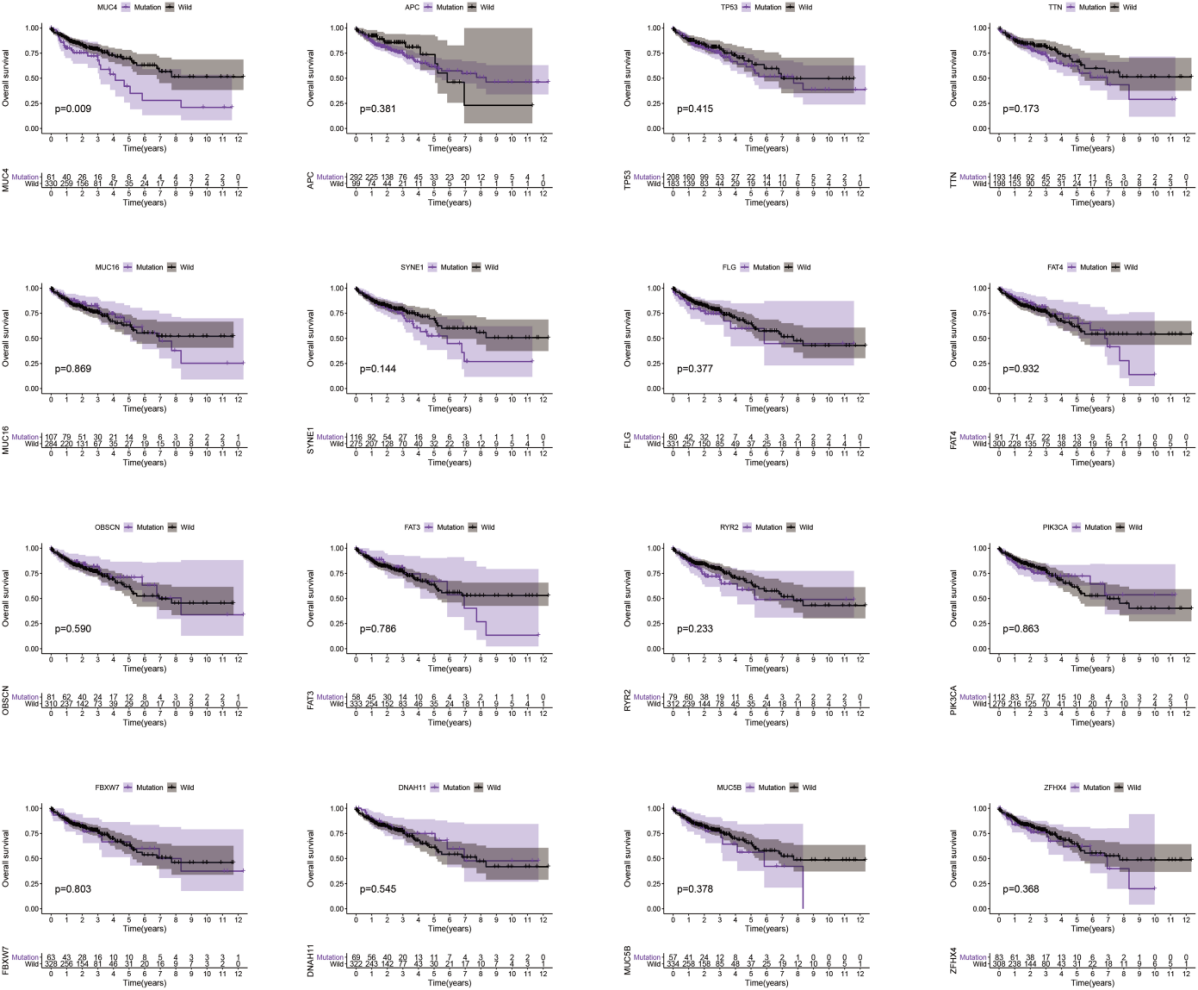
**MUC4 mutation is associated with clinical prognosis.** Kaplan-Meier survival analysis was used to determine survival curves that reflect the association between gene mutations and prognosis. The *p*-value is shown each plot.

**Figure 4:**
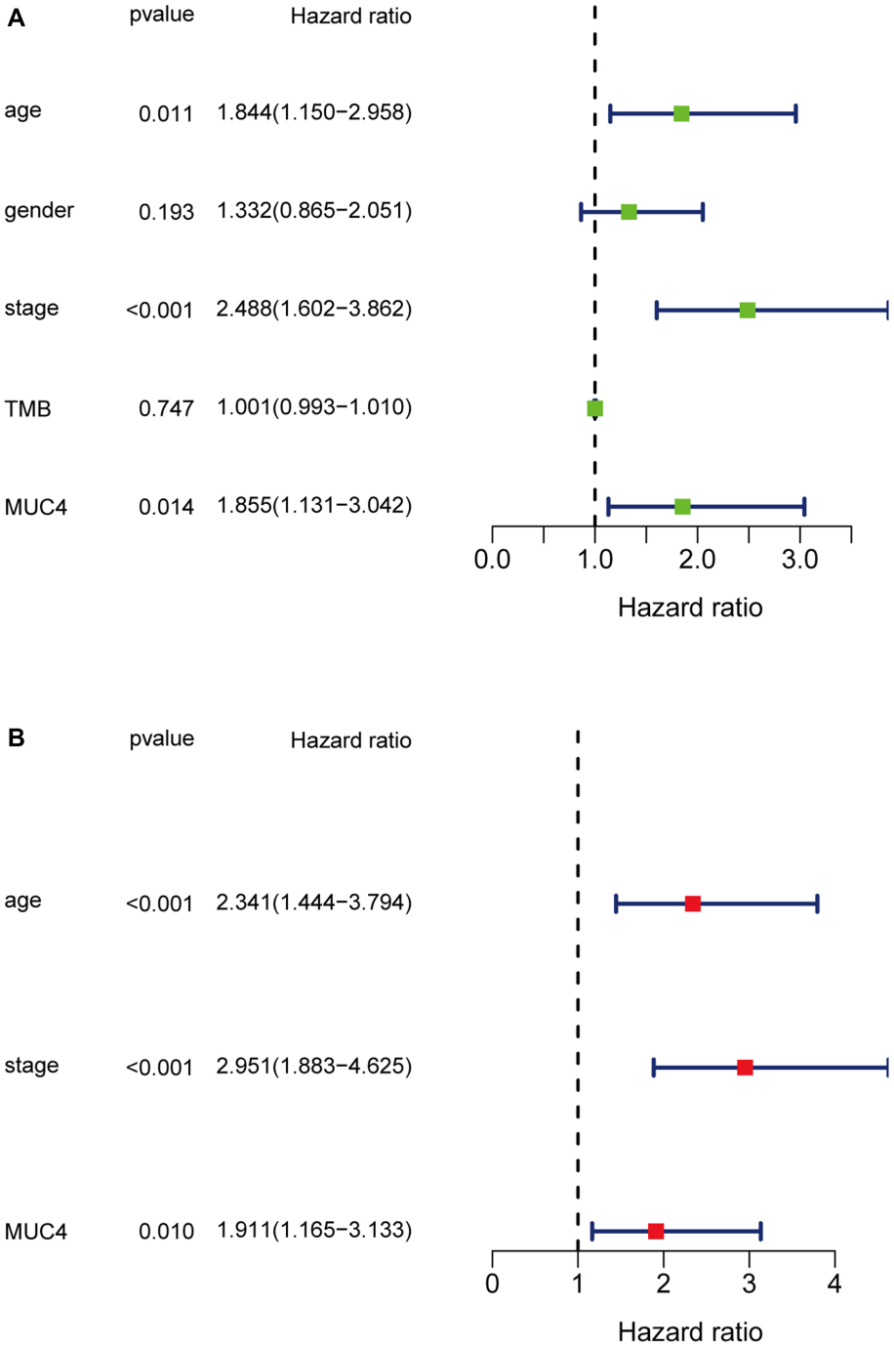
Univariate (**A**) and multivariate (**B**) overall survival analysis of colon cancer patients by the Cox proportional hazards model.

It is well known that TMB is associated with the relapse-free survival (RFS) in colon cancer [23]. Thus, considering the established association between 16 mutated genes and TMB, we speculate that these genes may be associated with clinical outcomes. For this purpose, patients from TCGA database were assigned to wild group and mutation group according to genes mutation status and Kaplan-Meier analysis was conducted with combining analysis of patient survival data. Our results demonstrated that only *MUC4* mutation was associated with a poor prognosis (*p* = 0.009) (Figure 3). Based on this funding, we aimed to further identify whether *MUC4* mutation is the independent prognostic factor for colon cancer using Cox regression analysis. As shown in Figure 4, With correction for common clinical information and TMB score, *MUC4* mutation remained significantly associated with overall survival of patients.

### Identification of enrichment pathways for patients with *MUC4* mutation

**Figure 5:**
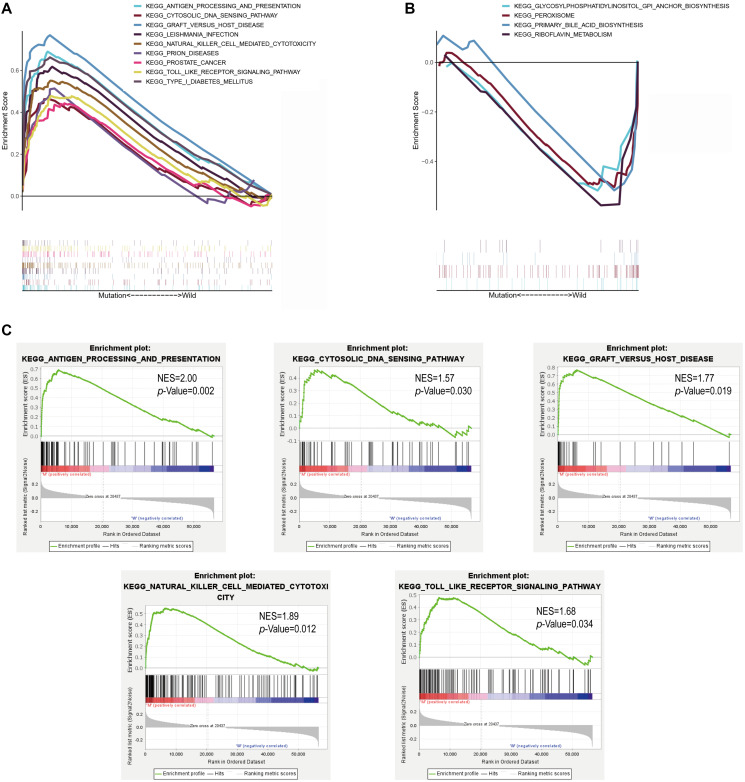
**MUC4 mutation is associated with immune-related pathways. Gene set enrichment analysis was performed with the TCGA.** (**A**) Multiple gene enrichment plot shows that a series of gene sets are enriched in the MUC4-mutant group. (**B**) Multiple gene enrichment plot shows that a series of gene sets are enriched in the wild-type MUC4 group. (**C**) Gene enrichment plots display that a series of immune-related gene sets are enriched in the MUC4-mutant group. NES, normalized enrichment score. The *p*-value is shown in each plot.

We next investigated the enrichment pathway associated with *MUC4* mutation. GSEA was performed, and the results showed that pathways were significantly enriched in the *MUC4* mutant group, including antigen processing and presentation, cytosolic DNA sensing pathway, prion diseases, graft versus host disease, type I diabetes mellitus, leishmania infection, toll like receptor signaling pathway, natural killer cell mediated cytotoxicity and prostate cancer (Figure 5A). Pathways that were significantly enriched in the *MUC4* wild-type group included glycosylphosphatidylinositol GPI anchor biosynthesis, peroxisome, primary bile acid biosynthesis, and riboflavin metabolism (Figure 5B). It is widely recognized that TMB is helpful to screen beneficiaries and predict the effect of immunotherapy. Considering the established association between *MUC4* mutation and TMB, thereby we speculate *MUC4* mutation may be correlated with immune response. As shown in Figure 5C, we observed that some immune-related pathways, including cytosolic DNA sensing pathway, antigen processing and presentation, natural killer cell mediated cytotoxicity, graft versus host disease and toll like receptor signaling pathway were enriched in *MUC4* mutation samples, while no immune response-related pathway was enriched in samples with wild-type *MUC4*.

### Tumor-infiltrating immune cells associated with *MUC4* mutation in colon cancer

**Figure 6:**
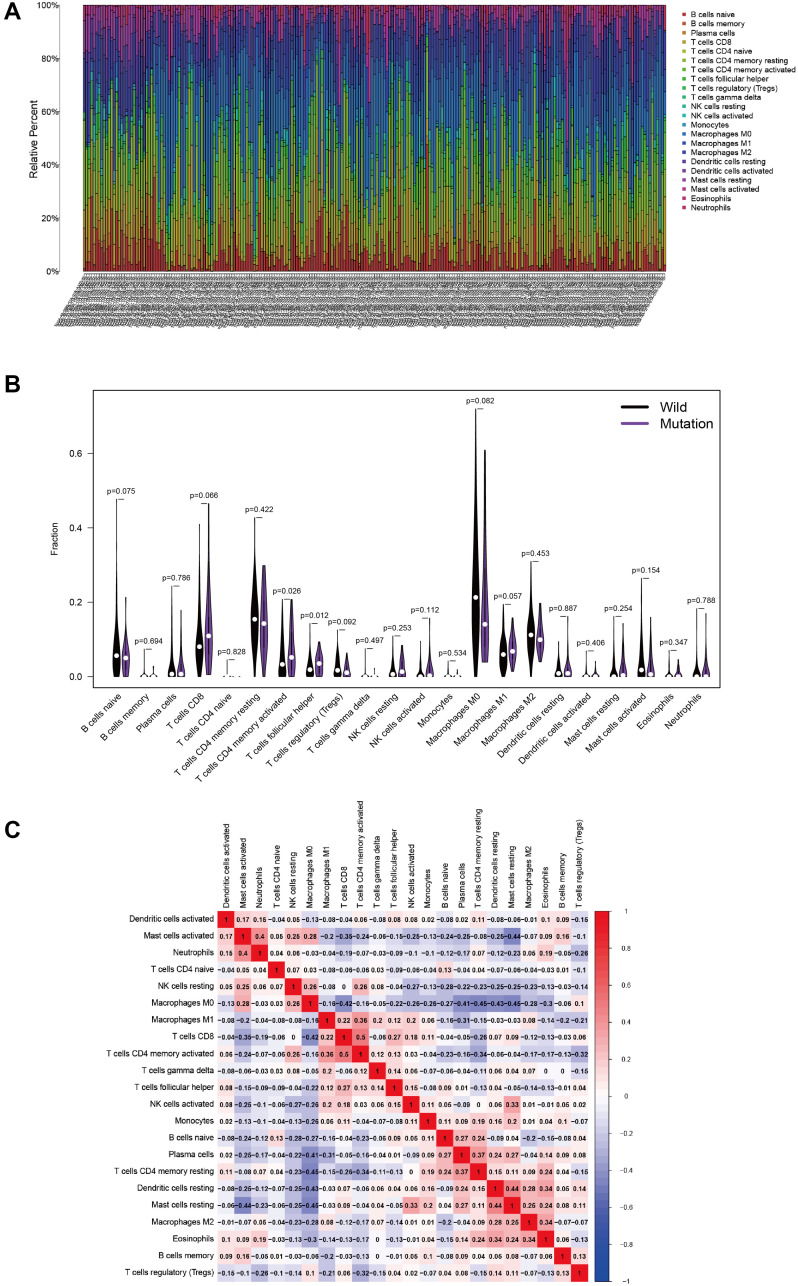
**MUC4 mutation is correlated with tumor-infiltrating immune cells.** (**A**) The stacked bar chart shows the distribution of 22 immune cells in each sample. (**B**) Violin plot displaying the differentially infiltrated immune cells between the MUC4-mutant groups and the wild-type MUC4 group. black represents the wild-type MUC4 group, and purple represents the MUC4-mutant group. The *p*-value is shown in the figure. (**C**) Correlation matrix of immune cell proportions. The red color represents a positive correlation, and the blue color represents a negative correlation.

Using CIBERSORT deconvolution algorithm, we first calculated the proportion of 22 immune cells for each sample in tumor tissue. The results revealed that the number of infiltrating immune cells changes greatly in different sample, and T cells and macrophages accounted for a relatively high proportion in the total samples (Figure 6A). Next, these samples were divided into *MUC4* wild group and *MUC4* mutation group to evaluate the situation of immune cell infiltration in the two groups. Compared to *MUC4* wild group, the infiltration proportion of follicular helper T cells and activated memory CD4 T cells were higher in MUC4 mutant group (Figure 6B). Finally, correlation analysis revealed that activated memory CD4 T cells had the strongest positive correlation with CD8 T cells, while they were negatively correlated with resting memory CD4 T cells and Tregs (regulatory T cells) (Figure 6C). Moreover, follicular helper T cells had the strongest positive correlation with CD8 T cells, and had the strongest negative correlation with M0 macrophages (Figure 6C).

## DISCUSSION

In our study, somatic mutation landscapes of colon cancer were characterized in 398 American samples and 304 Chinese samples. Subsequently, *MUC4* mutation was identified to be associated with TMB and patient clinical outcomes. Moreover, immune-related signaling pathways were significantly enriched in samples with *MUC4* mutation. Furthermore, *MUC4* mutant samples presented a higher infiltration proportion of follicular helper T cells and activated memory CD4 T cells, which is in line with previous established evidence that anti-tumor immune response was associated with these immune cells and pathways [24–26].

The membrane mucin *MUC4* is abundantly expressed in many epithelia and is overexpressed in some epithelial tumors [27, 28]. *MUC4* is known to play an anti-adhesive role by regulating ErbB2 and ErbB3 phosphorylation as a ligand/modulator of ErbB2 [29–31]. In cancer, *MUC4* upregulation contributed to tumor proliferation, apoptosis, invasiveness and metastasis in an ErbB2-dependent and ErbB2-independent manner, and multiple signaling pathways are involved in its regulatory mechanisms, such as the PI3-kinase/Akt pathway, gp130/STAT3 pathway and Erk pathway [32–34]. Specifically, *MUC4* mutation is also widely observed in pancreatic ductal adenocarcinoma and gastric cancer [35, 36]. In addition, Yang et al. reported that patients with *MUC4* mutation showed lower T stages and were related to patient prognosis in gastric cancer [36]. Colon cancer is a highly heterogeneous tumor involving several well-known gene mutations, including KRAS, BRAF, TP53 and PIK3CA, and *MUC4* is also reported as a frequently mutated gene in colon cancer [37, 38]. Here, *MUC4* mutation was identified to be associated with TMB and patient clinical outcomes. TMB is the total number of somatic cell mutations, and can also be defined as nonsynonymous mutations, and 1 to 2 neoantigens may be produced by every 150 nonsynonymous mutations [15]. These neoantigens can be recognized by the autoimmune system, thereby activating T cells and initiating the immune response [39, 40]. Thus, we speculated that *MUC4* mutation with a high TMB in colon cancer might drive the immune system to fight against tumor cells.

With detecting of peripheral blood samples in metastatic epithelial cancer, a recent study has demonstrated that mutations in the *MUC4* antigen can be recognized by memory T cells, indicating the existence of somatic mutations in the *MUC4* antigen during cancer progression [41]. In tumor immunity, CD4 T cells can activate cytotoxic T lymphocytes (CTLs) through a variety of mechanisms to maintain and strengthen the antitumor response of CTLs, while the presence of infiltrating Tregs may be detrimental to the host defense against the tumor [25, 42]. Specifically, it has been reported that the lymph nodes had an enhanced infiltration proportion of memory CD4 T cells in breast cancer. Tumor recurrence of renal cell carcinoma can be prevented by the memory immune effect of CD4 T cells [43, 44]. In our study, we also revealed *MUC4* mutant samples presented a higher infiltration proportion of activated memory CD4 T cells, and it was positively related with CD8 T cells and negatively with Tregs. Thus, we speculated that *MUC4* mutation might positively regulate CD4 and CD8 T cell while negatively regulate Tregs in colon cancer. Moreover, we also observed that the infiltration proportion of follicular helper T cells were higher in *MUC4* mutant group compared with *MUC4* wild group. Follicular helper T cells contribute to the formation of germinal centers of B cells, and enhanced activation and differentiation ability of B cells [45]. It has also been well confirmed that the antitumor response can be facilitated by inducing T follicular helper cell to activate B cells with immune checkpoint therapy in breast cancer murine models [46], and T follicular helper cells potently enhance the effector functions of CD8 T cells via an IL-21-dependent pathway in colorectal cancer [26]. Therefore, our results demonstrated that the changed tumor-infiltrating immune cells induced by *MUC4* contribute to the antitumor immunity of colon cancer.

This research has some limitations. Due to the lack of clinical data in ICGC database, we cannot determine whether *MUC4* mutation is also associated with prognosis and tumor immunity in Chinese patients. Moreover, tumor immunotherapy is a very complex topic, including immune cells, cytokines, immune microenvironment, tumor-related gene mutations and antigens, Etc; while this study is all informatics analyses and further experimental validations are needed.

In conclusion, *MUC4* mutation was associated with TMB and patient survival and immune pathway and antitumor immune response. It may have important clinical significance for immune therapy of colon cancer.

## MATERIALS AND METHODS

### Data acquisition

Transcriptome and somatic mutation and clinical data for US colon cancer patients was obtained from TCGA (http://portal.gdc.cancer.gov/projects). Somatic mutation data for Chinese patients was downloaded from ICGC (http://dcc.icgc.org/releases/current/ Projects). Data was extracted and organized in Perl so that it can be analyzed in R. Only patients with complete clinical data were included, excluding those patients with missing data such as sex, age, TNM stage and survival information.

### Definition of TMB in colon cancer

TMB was calculated as the total number of mutated bases per megabase, and only mutations that cause changes in amino acids were counted. The expression of TMB in each TCGA colon cancer sample was calculated by the TMB formula [15].

### Bioinformatic analysis

All bioinformatic analyses was performed by R software (v4.0.2). Genes with the top 30 mutation frequencies in TGCA and IGGC databases were respectively extracted by Perl. The R package "GenVisR" was used to visualize the mutations of these genes [47]. These genes were intersected to obtain genes with high mutation frequency in both databases by R package "venn". The relationship between these intersection mutated genes and TMB was assessed and visualized using R package "ggpubr". GSEA analysis was performed using *MUC4* mutation and expression matrix data in GSEA software (v4.1.0) [48]. “c2.cp.kegg.v7.2.symbols.gmt” was selected as the gene sets database. Normalized enrichment score (NES) was calculated by setting the permutations value to 1000, and the FDR *p*-value <0.05 was used to identify significant enrichment pathways. CIBERSORT is a computational method for assessing the proportion of 22 immune cells in tumor tissue based on transcriptome data [49]. A matrix data of immune cell proportion for each tumor sample was obtained using CIBERSORT deconvolution algorithm with setting the filter condition to *p* < 0.05. The matrix data visualization was performed by R package "corrplot". TCGA samples were assigned to wild group and mutation group based on *MUC4* status. Difference analysis of infiltrating immune cells between the two groups was performed by R package "limma" and visualized by R package "vioplot".

### Statistical analysis

R (v4.0.2) was used for statistical analyses. Survival curves were analyzed with Kaplan-Meier survival analysis and evaluated using the log-rank test. Identification of prognosis risk factor was performed by univariate and multivariate Cox regression analyses. The correlation between mutant genes and TMB was analyzed by the Mann-Whitney *U* test. For all comparisons, a two-tailed *p*-value <0.05 was considered statistically significant.
